# Acute Responses to Creatine Monohydrate and L-Arginine, Alone and Combined, on Repeated-Sprint Power, Countermovement Jump Height, and Stroop Reaction Time in Recreationally Active Men: A Randomized Double-Blind Crossover Trial

**DOI:** 10.3390/metabo16090688

**Published:** 2026-09-18

**Authors:** Özgür Eken, Halil Uçar, Ahmet Kurtoğlu, Murat Ozan, Yusuf Buzdağlı, Mehmet Söyler, Monira I. Aldhahi

**Affiliations:** 1 Department of Physical Education and Sport Teaching, Faculty of Sport Sciences, İnönü University, 44000 Malatya, Türkiye; ozgur.eken@inonu.edu.tr (Ö.E.); halil.ucar@inonu.edu.tr (H.U.); 2 Department of Coaching Education, Faculty of Sport Science, Bandirma Onyedi Eylul University, 10200 Balikesir, Türkiye; 3 Department of Physical Education and Sport Teaching, Faculty of Sport Sciences, Atatürk University, 25000 Erzurum, Türkiye; murat.ozan@atauni.edu.tr; 4 Department of Coaching Education, Faculty of Sport Sciences, Erzurum Technical University, 25000 Erzurum, Türkiye; yusuf.buzdagli@erzurum.edu.tr; 5 Vocational School of Social Sciences, Çankırı Karatekin University, 18200 Çankırı, Türkiye; mehmetsoyler@karatekin.edu.tr; 6 Department of Rehabilitation Sciences, College of Health and Rehabilitation Sciences, Princess Nourah bint Abdulrahman University, P.O. Box 84428, Riyadh 11671, Saudi Arabia

**Keywords:** creatine monohydrate, L-arginine, repeated-sprint performance, countermovement jump, Stroop reaction time, crossover trial

## Abstract

Background/Objectives: Creatine monohydrate (CR) and L-arginine (LA) are widely used ergogenic supplements, but whether acute co-ingestion provides additional performance or task-specific cognitive benefits beyond either supplement alone remains uncertain. This study compared placebo, CR, LA, and LA+CR on repeated-sprint power, countermovement jump (CMJ) height, and Stroop reaction time (RT). Methods: In this randomized, double-blind, four-period crossover trial, 18 recreationally active men completed placebo (CG), CR (0.3 g·kg^−1^), LA (6 g), and LA+CR conditions separated by at least 72 h (prospectively registered at ClinicalTrials.gov: NCT07548541). Testing began 60 min after ingestion with the Stroop test, followed by CMJ and the Running-Based Anaerobic Sprint Test (RAST). Co-primary outcomes were RAST average power (AP), peak power (PP), CMJ height, and incongruent Stroop median RT. Results: Condition effects were observed for AP, PP, minimum power, CMJ height, and Stroop RT (all *p* ≤ 0.011), but not fatigue index or Stroop accuracy. All four co-primary omnibus effects remained significant after Holm correction (all pHolm < 0.001). LA+CR produced higher PP than CG (MD 99.01 W; *p* < 0.001), LA (MD 51.29 W; *p* = 0.002), and CR (MD 46.71 W; *p* < 0.001), and greater CMJ height than all comparison conditions (all *p* < 0.001). LA+CR also produced lower Stroop RT than CG (MD 0.089 s; *p* < 0.001) and CR (*p* = 0.041), but not LA (*p* = 0.271). For AP, LA+CR exceeded CG and CR, but not LA after Bonferroni adjustment. In a secondary 2 × 2 factorial decomposition, no LA×CR interaction for a co-primary outcome survived Holm correction (all pHolm ≥ 0.104). Conclusions: Acute LA+CR produced outcome-specific improvements in peak sprint power and CMJ and selectively reduced Stroop RT, but did not establish biological synergy. Interpretation should remain exploratory given the small male-only sample, unmodelled period/sequence effects, and possible creatine-related carryover.

## 1. Introduction

Nutritional supplementation is widely used to support exercise performance, but the evidential strength varies substantially across compounds and dosing strategies [1]. Creatine monohydrate (CR) and L-arginine (LA) have each been studied as ergogenic aids, with stronger and more consistent evidence for multi-day creatine supplementation than for acute LA supplementation [2,3,4,5,6,7]. Multi-ingredient strategies are increasingly common, yet combining two plausible ergogenic agents does not necessarily yield additive or synergistic effects [8].

LA is a conditionally essential amino acid and a substrate for nitric oxide (NO) synthesis, with potential effects on vascular tone and exercise hyperemia [9,10]. LA also participates in pathways related to guanidinoacetate/creatine and agmatine metabolism [10,11,12,13]. However, the ergogenic response to oral LA is heterogeneous and appears sensitive to dose, bioavailability, participant characteristics, and exercise modality [14,15]. NO signaling can influence skeletal-muscle glucose metabolism, mitochondrial redox biology, calcium-dependent contractile processes, and muscle force production, but these effects are context dependent rather than uniformly ergogenic [16,17,18,19,20,21,22].

Creatine is concentrated in skeletal muscle and contributes to rapid ATP buffering through the phosphocreatine (PCr) system during high-intensity exercise [23,24]. Established loading protocols increase intramuscular total creatine and PCr and can improve repeated high-intensity performance [25,26,27,28]. In contrast, the physiological basis for a performance effect from a single pre-exercise creatine bolus is less certain, because conventional muscle-loading protocols require repeated dosing over several days [2,27,28]. Creatine has also been studied in relation to brain energetics and cognition, although results in healthy young adults are mixed and may depend on baseline creatine status or metabolic stress [29,30,31,32,33].

Direct evidence on combined CR and LA administration is sparse [34,35]. This creates a reasonable empirical question, but the novelty is primarily treatment-combination and outcome-specific rather than the discovery of a new biological pathway.

A mechanistic rationale for co-administration can be proposed because LA may influence NO-related vascular responses and CR supports cellular energy buffering [36]. However, current evidence does not establish that acute LA ingestion increases skeletal-muscle or brain creatine uptake. Neurovascular coupling and exercise-fatigue physiology are complex processes [37,38], so any interaction between LA and CR should be tested empirically rather than assumed from pathway complementarity.

The brain has high energetic requirements, and neuronal function depends on tightly regulated ATP production and buffering [39,40]. Creatine transport and the cerebral creatine–phosphocreatine system may contribute to neuronal energy homeostasis [41,42]. These observations provide a rationale for including a cognitive task, but a single Stroop outcome cannot establish a broad cognitive-enhancement effect [43].

Accordingly, this study compared the acute effects of placebo, CR, LA, and LA+CR on repeated-sprint power, CMJ height, and incongruent Stroop reaction time in recreationally active men. The four co-primary outcomes listed in the registry were RAST AP, RAST PP, CMJ height, and incongruent Stroop median RT. We hypothesized that LA+CR would produce more favorable acute responses than either single-agent condition or placebo; mechanistic interpretations were considered secondary and hypothesis-generating.

## 2. Materials and Methods

### 2.1. Study Design

**Figure 1:**
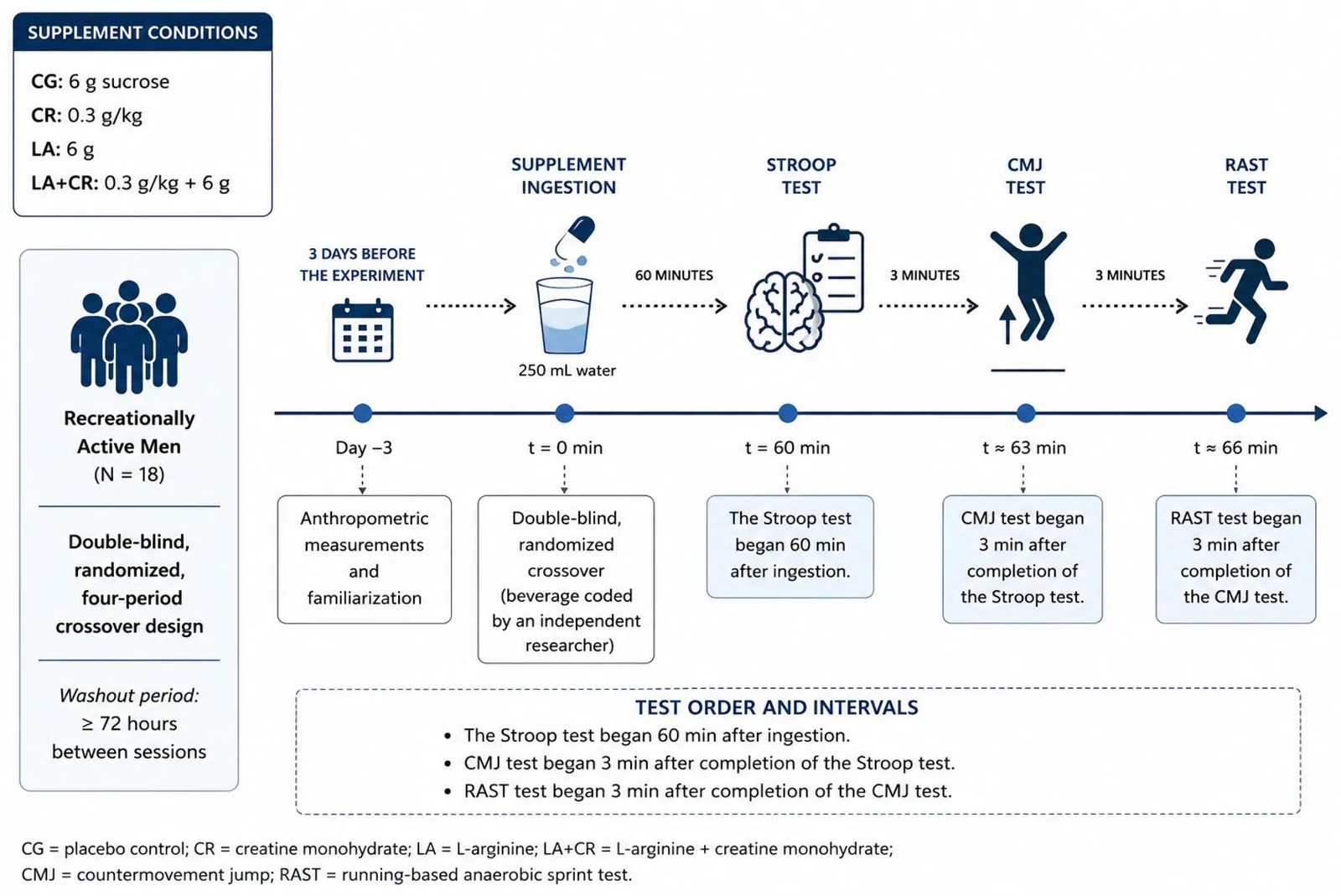
Schematic overview of the randomized, double-blind, four-period crossover protocol. Participants were scheduled to complete all four conditions (CG, LA, CR, LA+CR), with ≥72 h between experimental sessions.

This study employed a randomized, double-blind, four-period, four-treatment crossover design in which each participant served as his own control. Participants were assigned to complete four experimental conditions in counterbalanced order: placebo (CG), CR, LA, and combined LA+CR. Treatment order was generated using a computer-based Latin-square approach (Random.org). The trial was prospectively registered at ClinicalTrials.gov (NCT07548541). The registration record was first submitted on 14 April 2026 and first posted on 23 April 2026, before the actual study start on 24 April 2026. According to the current registry record, primary completion was 15 May 2026, study completion was 16 May 2026, and the record was verified in August 2026. Sessions were conducted at the same time of day (±30 min) and separated by at least 72 h. This interval standardized scheduling but cannot be assumed to eliminate creatine-related carryover. Participants and outcome assessors were blinded to treatment identity. The study was approved by the İnönü University Ethics Committee (Approval No. 2025/6879; 25 March 2025) and conducted in accordance with the Declaration of Helsinki. Written informed consent was obtained from all participants before study procedures. A schematic overview of the experimental protocol is presented in Figure 1. Reporting was prepared with reference to CONSORT 2025 and crossover-specific reporting recommendations.

### 2.2. Participants

An a priori power analysis (G*Power 3.1) indicated that 13 participants would be sufficient to detect a medium within-subject effect (f = 0.25) using repeated-measures ANOVA at α = 0.05 and 80% power. Because four co-primary outcomes were prespecified, this calculation should be interpreted as a general design calculation rather than multiplicity-adjusted power for every co-primary outcome. Eligible participants were healthy, recreationally active men aged ≥18 years. Inclusion criteria were: (i) ≥2 years of regular participation in organized sport; (ii) no known orthopedic, neurological, cardiovascular, pulmonary, or metabolic disorders; and (iii) no use of anabolic steroids or performance-enhancing drugs within the previous 3 months. Participants using psychoactive or stimulant substances during the study were excluded.

### 2.3. Experimental Procedures

Each participant attended up to four experimental sessions at the same time of day (±30 min), with at least 72 h between sessions. During each session, the Stroop test was performed first; the CMJ began 3 min after completion of the Stroop test, and the RAST began 3 min after completion of the CMJ. This fixed within-session order standardized comparisons but did not eliminate potential practice, priming, or task-order effects across repeated visits.

A familiarization session and anthropometric measurements were conducted 72 h before the first performance session. The four co-primary outcomes listed in the registry were RAST peak power (PP), RAST average power (AP), CMJ height, and incongruent Stroop median reaction time (RT). Secondary outcomes were RAST minimum power (MP), fatigue index (FI), and Stroop accuracy. The family-wise Type I error rate across the four co-primary omnibus tests was controlled with the Holm step-down procedure; post hoc pairwise comparisons were Bonferroni-adjusted within outcome.

### 2.4. Data Collection Procedures

#### 2.4.1. Anthropometric Measurements

Participants’ ages were recorded using official identification. Body mass was measured to the nearest 0.01 kg while participants wore shorts and stood barefoot. Height was measured barefoot in an upright position, with heels together and the head in the Frankfurt plane, using a Soehnle ultrasonic height measurer (accuracy ± 1 mm). Body mass index (BMI) was calculated as body mass (kg)/height^2^ (m^2^).

#### 2.4.2. Supplementation Protocol

Participants arrived at the laboratory 90 min before testing. CR was administered at 0.3 g·kg^−1^ as a single acute bolus. This amount is similar to the conventional total daily amount used during creatine loading, but standard loading protocols divide approximately 0.3 g·kg^−1^·day^−1^ across several doses for 5–7 days; therefore, the present single-bolus protocol should not be interpreted as an established acute loading strategy [44]. LA was administered at 6 g, and the combined condition contained CR 0.3 g·kg^−1^ plus LA 6 g in the same beverage [45]. Each active condition was diluted in 250 mL water, whereas the placebo control (CG) contained 6 g sucrose in 250 mL water. The placebo was matched for volume, flavor, and appearance but was not mass-matched or iso-osmotic with the active conditions. The Stroop test began 60 min after ingestion; CMJ and RAST were performed sequentially thereafter and were therefore assessed later than 60 min post-ingestion. Participants were questioned about gastrointestinal discomfort, dizziness, and headache at 0, 60, and 120 min and again 24 h after intake.

All drinks were flavored with a neutral citrus aroma and colored uniformly. An independent researcher prepared coded (A–D) solutions and administered them according to the randomized treatment order. Participants and data collectors were unaware of the treatment codes; blinding effectiveness was not formally assessed.

To standardize dietary intake, participants were provided with a diet plan containing approximately 60% carbohydrate, 25% fat, and 15% protein beginning 72 h before testing. Participants maintained 3-day weighed food diaries, and compliance with dietary and caffeine restrictions was monitored using dietary analysis software. A list of caffeine-rich foods and beverages to be avoided—including coffee, tea, mate, energy drinks, cola, chocolate beverages, and chocolate—was provided to all participants.

#### 2.4.3. Running-Based Anaerobic Sprint Test (RAST)

The RAST consisted of six 35 m maximal sprints separated by 10 s of recovery. Participants performed alternating-direction sprints, and sprint times were recorded using a Smart Speed telemetric timing system (Fusion Sport, Brisbane, Australia). Before the test, participants completed a standardized 10 min warm-up consisting of 5 min of self-paced jogging, dynamic stretching, and several 10–15 m accelerations. Derived outcomes were peak power (PP, W), minimum power (MP, W), average power (AP, W), and fatigue index (FI, %). Power for each sprint was calculated as body mass (kg) × distance^2^ (m^2^)/time^3^ (s^3^) [46]. Relative power and relative work were not analyzed.

#### 2.4.4. Countermovement Jump Test (CMJ)

Participants stood with feet shoulder-width apart and hands fixed on the hips (akimbo position), performed a rapid countermovement, and then jumped vertically with maximal effort. CMJ height was measured using an Optojump optical system (Microgate S.r.l, Bolzano, Italy). Three maximal jumps were performed with 30 s between attempts; the best jump height was used for the co-primary CMJ outcome (ICC = 0.92; CV = 2.8%). The CMJ was performed 3 min after completion of the Stroop test.

#### 2.4.5. Stroop Color–Word Test (SCWT)

The Stroop Color–Word Test (SCWT) assesses interference control by requiring responses to stimulus color in the presence of congruent or incongruent word information [47]. In the present study, the task was used as a specific measure of incongruent response speed and accuracy; results should not be generalized to overall cognitive function.

The computerized SCWT was administered using PsychLab version 3.2 in English. Participants reported sufficient familiarity with the color words used (red, green, blue, and yellow). Each session consisted of 40 congruent, 40 incongruent, and 40 neutral stimuli. Reaction times <200 ms or >3 SD above the participant-specific mean were excluded. The co-primary cognitive outcome was median RT in the incongruent condition; accuracy (%) was secondary. Use of an English-language task in a Turkish-speaking sample and the high accuracy values were considered measurement limitations.

### 2.5. Statistical Analysis

All analyses were performed in R version 4.3 (R Foundation for Statistical Computing, Vienna, Austria) via RStudio. The primary efficacy analysis used one-way repeated-measures ANOVA with condition (CG, LA, CR, LA+CR) as the within-subject factor. Residual distributions were assessed using Shapiro–Wilk tests, and sphericity was evaluated with Mauchly’s test; Greenhouse–Geisser correction was applied when sphericity was violated. The four omnibus *p* values for the co-primary outcomes (AP, PP, CMJ, and Stroop RT) were Holm-adjusted as one family. For outcomes with a significant omnibus effect, pairwise comparisons were Bonferroni-adjusted within that outcome. The 95% confidence intervals reported for pairwise contrasts are unadjusted, whereas the tabulated *p* values are Bonferroni-adjusted; therefore, CI exclusion of zero and adjusted-*p* significance may differ. Partial eta squared (ηp^2^) is reported for omnibus effects, and within-subject standardized mean differences (d_x_ = Mdiff/SDdiff) are reported for pairwise comparisons. To address the 2 × 2 factorial treatment structure, a secondary within-participant contrast analysis estimated the LA main effect as [(LA − CG) + (LA+CR − CR)]/2, the CR main effect as [(CR − CG) + (LA+CR − LA)]/2, and the LA×CR interaction as LA+CR − LA − CR + CG. Each contrast was tested against zero, with Holm adjustment across the four co-primary outcomes separately within the LA, CR, and interaction families. The retained analysis dataset contained treatment-condition labels but not randomized-sequence or period identifiers; therefore, period, sequence, participant-within-sequence, treatment-by-period, and first-order carryover effects could not be estimated. The ≥72-h washout cannot be assumed to eliminate creatine-related carryover, so the factorial analysis is considered a secondary sensitivity analysis and no claim of absent carryover is made. Statistical significance was set at α = 0.05.

## 3. Results

### 3.1. Participant Characteristics

**Figure 2:**
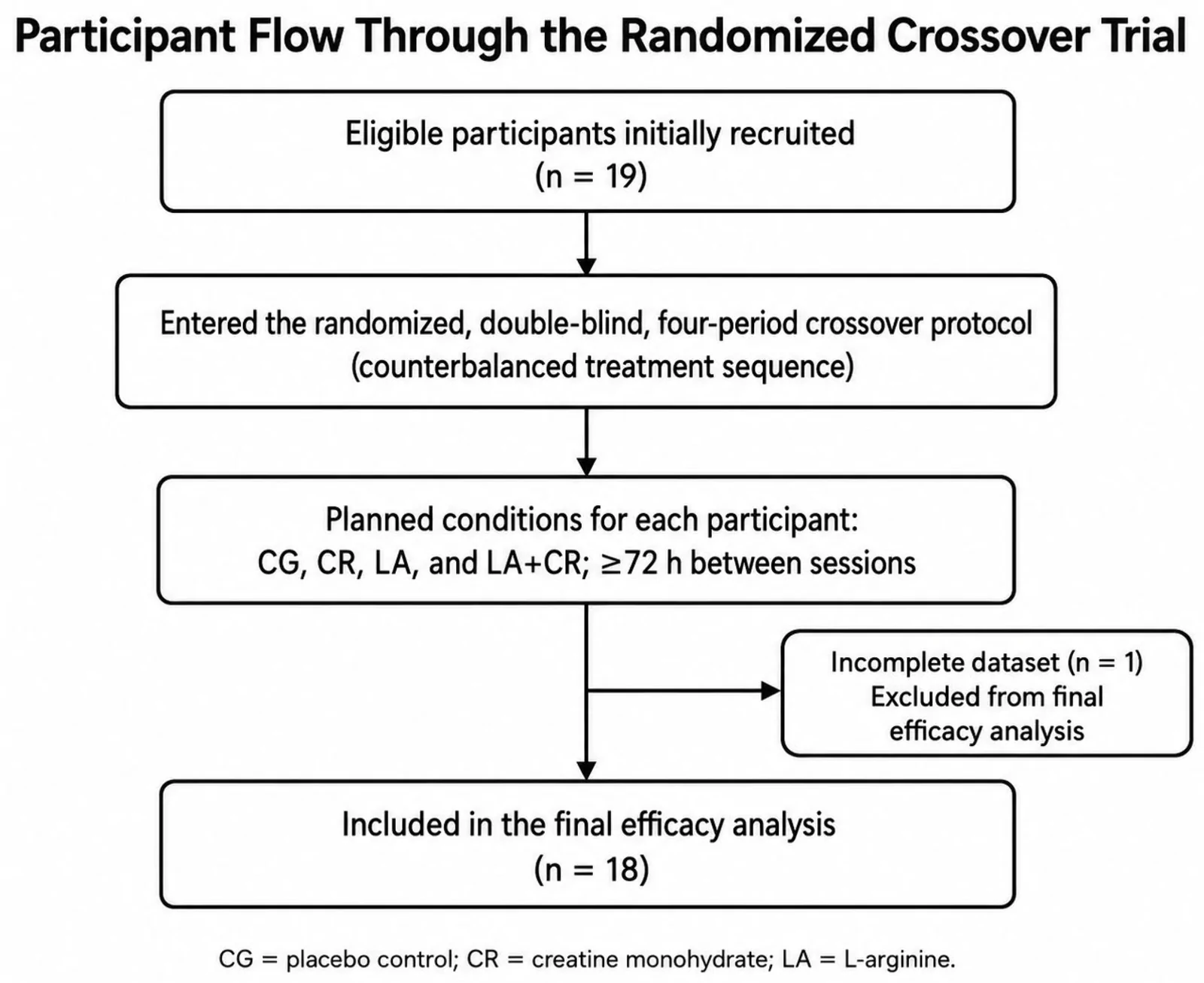
Participant flow through the randomized, double-blind, four-period crossover trial. CG = placebo control; CR = creatine monohydrate; LA = L-arginine.

Nineteen participants were initially recruited. One participant had an incomplete dataset and was excluded from the final efficacy analysis, leaving 18 participants for analysis. Participant disposition is summarized in Figure 2.

Table 1 summarizes the characteristics of the 18 analyzed participants. Mean age was 21.4 ± 2.1 years, mean height was 177.3 ± 4.8 cm, mean body mass was 71.8 ± 10.1 kg, and mean BMI was 22.8 ± 2.9 kg/m^2^. Because CR was dosed by body mass, individual CR doses ranged from 16.2 to 26.1 g.

No gastrointestinal discomfort, dizziness, or headache was reported during the 0-, 60-, and 120-min assessments or at the 24-h follow-up in any treatment condition.

### 3.2. Preliminary Checks

Shapiro–Wilk tests did not provide evidence against normality of the model residuals for the reported outcomes (all W ≥ 0.974, all *p* > 0.13). Mauchly’s test indicated sphericity violations for AP (W = 0.322, *p* = 0.002), MP (W = 0.232, *p* < 0.001), FI (W = 0.210, *p* < 0.001), and Stroop RT (W = 0.318, *p* = 0.002); Greenhouse–Geisser corrections were therefore applied. Sphericity was not rejected for PP (W = 0.527, *p* = 0.055), CMJ (W = 0.581, *p* = 0.102), or Stroop accuracy (W = 0.932, *p* = 0.945).

### 3.3. Descriptive Statistics

Table 2 reports the unadjusted descriptive statistics for each condition. LA+CR had the highest mean AP, PP, and CMJ height and the lowest mean Stroop RT, whereas LA had the highest mean MP. FI means ranged from 21.66% to 24.74%, and Stroop accuracy was high in every condition (approximately 97–98%).

### 3.4. Omnibus ANOVA Results

The omnibus repeated-measures analyses showed condition effects for AP, PP, MP, CMJ height, and Stroop RT (all *p* ≤ 0.011; Table 3). No statistically significant condition effect was detected for FI or Stroop accuracy (both *p* > 0.30). For the four co-primary outcomes (AP, PP, CMJ, and Stroop RT), all omnibus effects remained statistically significant after Holm correction (all pHolm < 0.001). Effect magnitudes and precision are considered alongside the adjusted *p* values.

### 3.5. Running-Based Anaerobic Sprint Test

A significant main effect of condition was observed for AP [F(1.92, 32.70) = 26.56, *p* < 0.001, ηp^2^ = 0.61]. Bonferroni-adjusted pairwise comparisons (Table 4) revealed that LA+CR produced significantly higher AP than CG (*p* < 0.001) and CR (*p* = 0.016), while the LA+CR vs. LA comparison approached but did not reach significance (*p* = 0.056). Both LA and CR alone also exceeded CG (both *p* ≤ 0.002) but did not differ from each other (*p* = 1.000). For PP [F(3, 51) = 24.35, *p* < 0.001, ηp^2^ = 0.59], LA+CR yielded the highest values, significantly exceeding all other conditions (all *p* ≤ 0.003).

For MP [F(1.89, 32.15) = 5.37, *p* = 0.011, ηp^2^ = 0.24], LA had the highest mean value and was higher than CG (Bonferroni-adjusted *p* < 0.001) and LA+CR (*p* = 0.027). FI did not show a statistically significant condition effect [F(1.71, 29.03) = 1.22, *p* = 0.306, ηp^2^ = 0.07]; no FI pairwise comparisons were interpreted.

For PP, LA+CR was higher than each of the other conditions, with MDs ranging from 46.71 to 99.01 W. For AP, LA+CR was higher than CG and CR, whereas the LA+CR versus LA comparison did not meet the Bonferroni-adjusted significance threshold (MD = 14.02 W, *p* = 0.056). For MP, LA was higher than CG and LA+CR. These contrasts describe distinct outcome patterns and do not by themselves establish biological synergy or antagonism between CR and LA.

**Figure 3:**
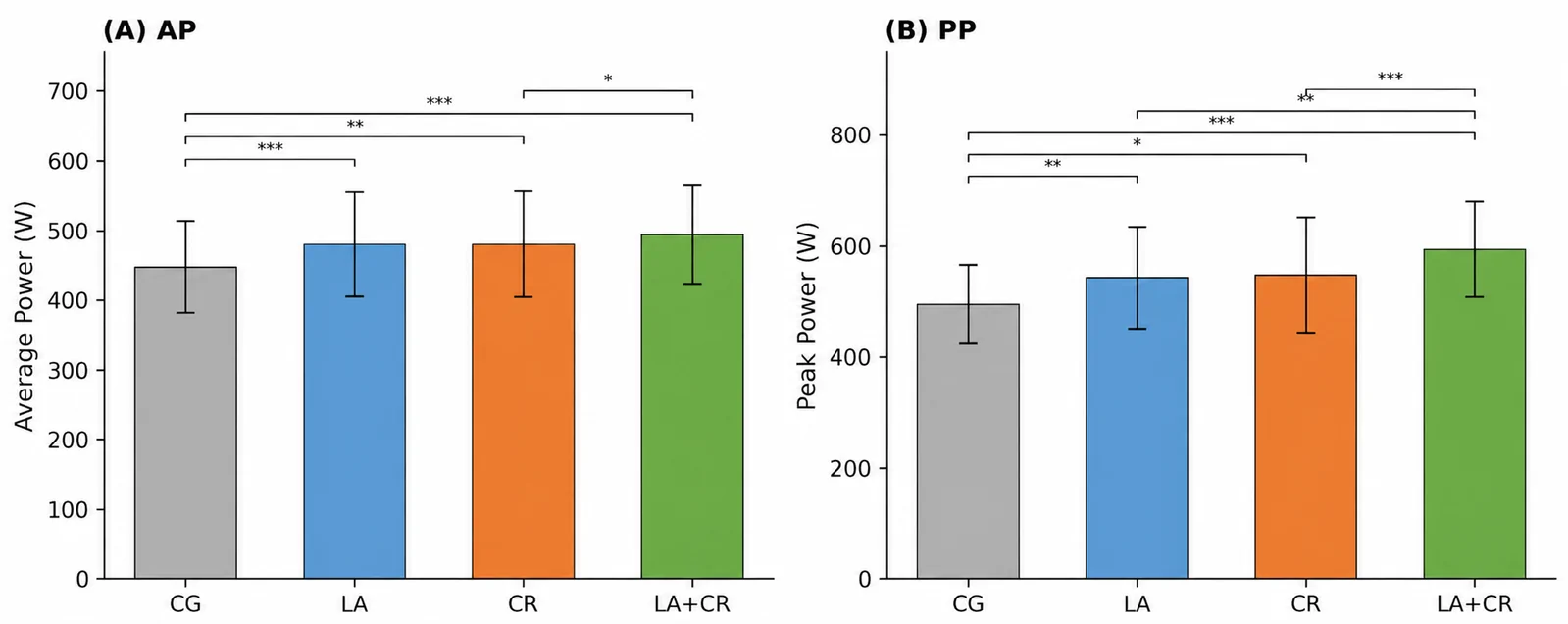

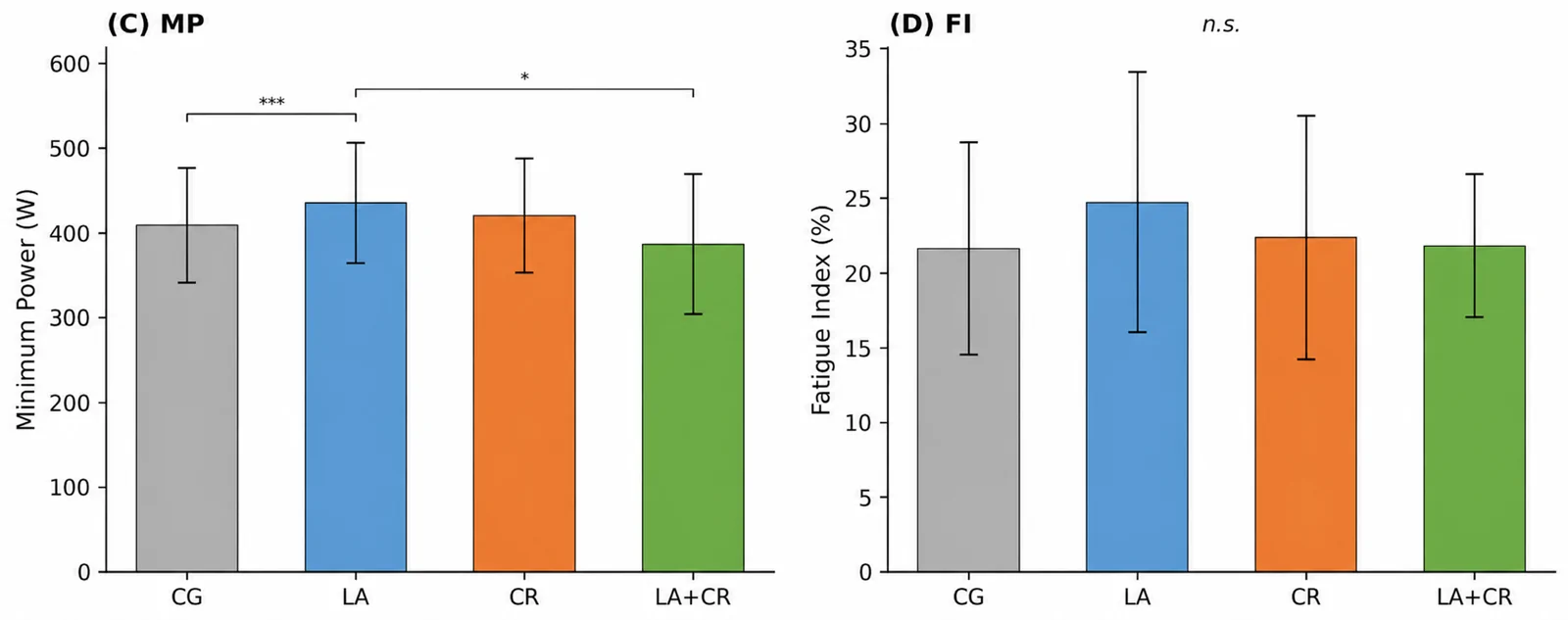
RAST results across conditions (N = 18). (**A**) Average power; (**B**) Peak power; (**C**) Minimum power; (**D**) Fatigue index. Bars represent mean ± SD. * *p* < 0.05, ** *p* < 0.01, *** *p* < 0.001 (Bonferroni-corrected). n.s. = not significant.

Figure 3 displays the condition means for AP, PP, MP, and FI. The inferential comparisons are reported in Table 3 and Table 4; visual overlap of SD error bars should not be used as a substitute for the within-subject statistical comparisons.

### 3.6. Stroop Color–Word Test

A significant main effect of condition was found for incongruent median RT [F(1.95, 33.21) = 15.84, *p* < 0.001, ηp^2^ = 0.48]. CG showed the slowest RT (0.626 ± 0.105 s), while LA+CR yielded the fastest (0.537 ± 0.080 s). All three active conditions produced significantly faster RTs than CG (all *p* ≤ 0.006; Table 5). LA+CR was also significantly faster than CR (*p* = 0.041), but did not differ from LA (*p* = 0.271). Accuracy did not differ across conditions [F(3, 51) = 1.01, *p* = 0.396, ηp^2^ = 0.06]; post hoc tests were not performed.

The largest RT contrast was LA+CR versus CG (MD = 0.089 s; d_x_ = 1.38; Bonferroni-adjusted *p* < 0.001), equivalent to a 14.2% lower mean RT. LA+CR also differed from CR (*p* = 0.041) but not from LA (*p* = 0.271). Accuracy did not show a significant condition effect; however, the uniformly high accuracy values may have limited sensitivity to detect a speed–accuracy trade-off.

**Figure 4:**
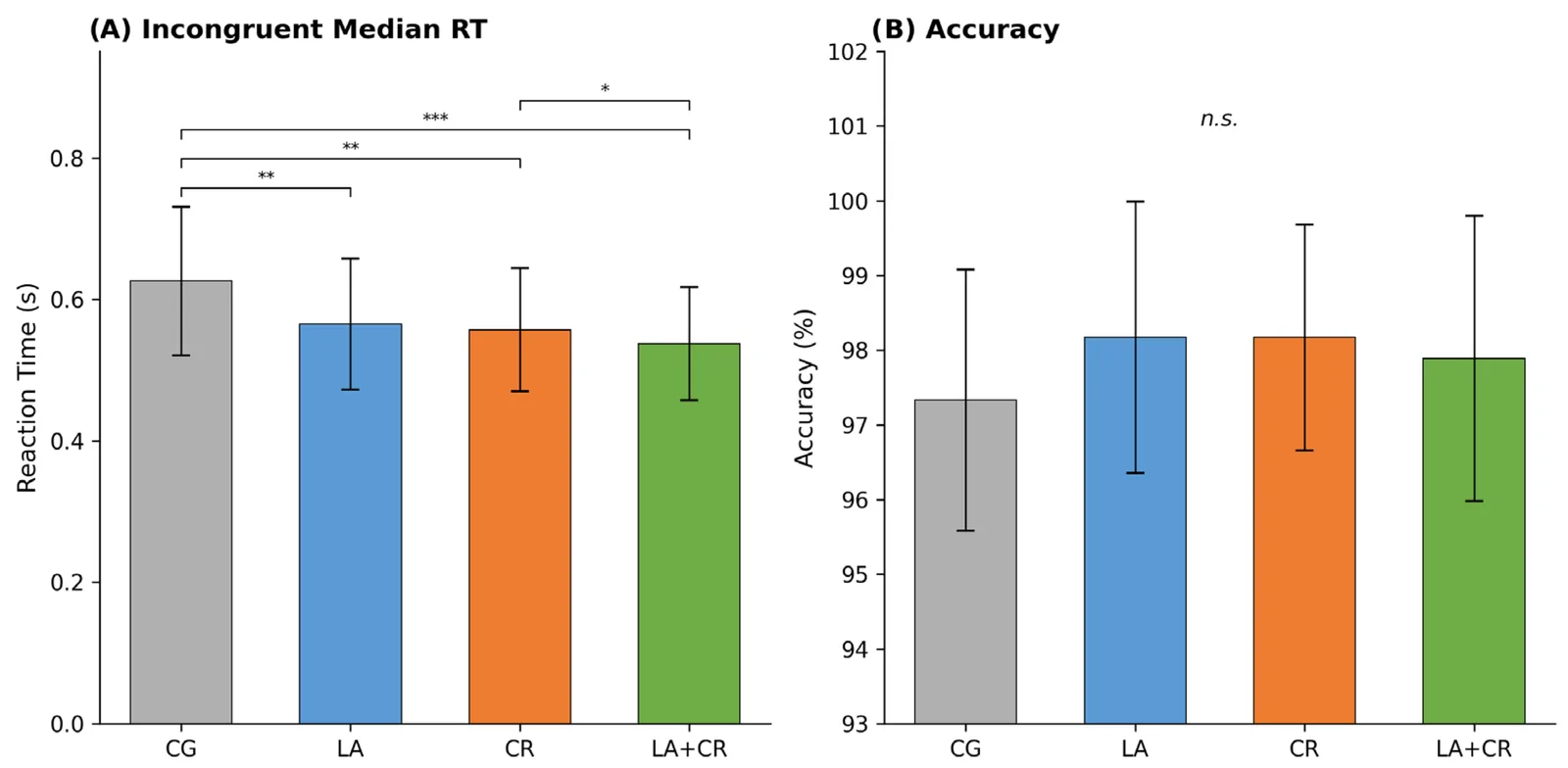
Stroop Color–Word Test results (N = 18). (**A**) Incongruent median RT; (**B**) Accuracy. Bars represent mean ± SD. * *p* < 0.05, ** *p* < 0.01, *** *p* < 0.001 (Bonferroni-corrected). n.s. = not significant.

Figure 4 shows lower mean incongruent RTs in all active conditions relative to CG, with LA+CR having the lowest mean RT. Accuracy remained high across conditions, but the narrow range and ceiling-level values limit strong inferences about speed–accuracy trade-offs.

### 3.7. Countermovement Jump

A significant main effect of condition was found for CMJ height [F(3, 51) = 85.36, *p* < 0.001, ηp^2^ = 0.83]. LA+CR produced the highest jump height (40.64 ± 3.88 cm), significantly exceeding CG, LA, and CR (all *p* < 0.001; Table 6). Both LA and CR also exceeded CG (both *p* < 0.001), but did not differ from each other (*p* = 0.983).

LA+CR produced a 6.72 cm higher mean CMJ than CG and was also higher than LA and CR (all Bonferroni-adjusted *p* < 0.001). LA and CR were each higher than CG, but did not differ from each other (*p* = 0.983). In the secondary factorial decomposition, however, the LA×CR interaction for CMJ did not remain statistically significant after correction (pHolm = 0.243; Table 7); the condition-wise pattern therefore does not establish additivity or synergy.

**Figure 5:**
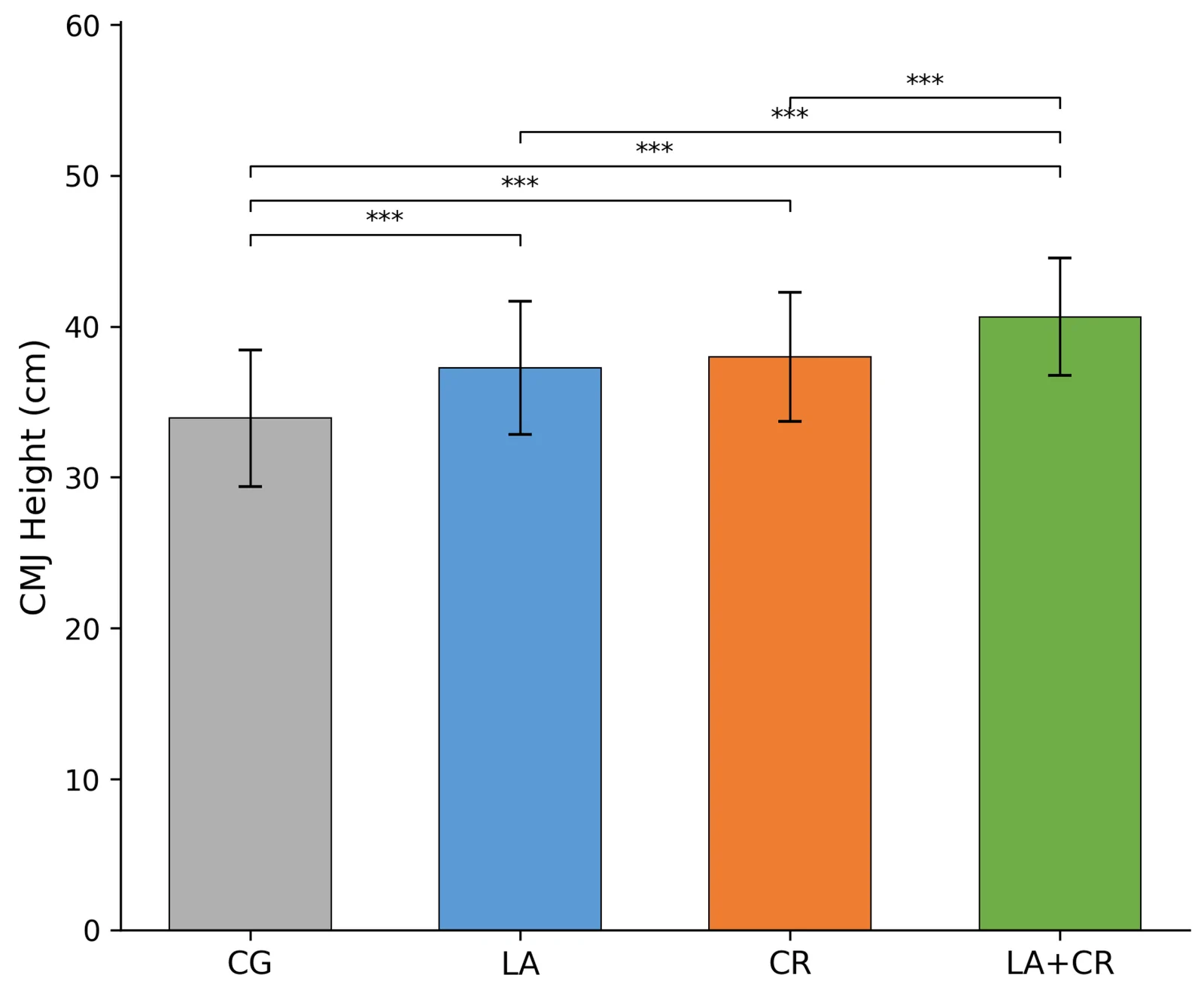
Countermovement jump height across conditions (N = 18). Bars represent mean ± SD. *** *p* < 0.001 (Bonferroni-corrected).

Figure 5 shows the condition means for CMJ height. The inferential pairwise comparisons are provided in Table 6.

### 3.8. Factorial 2 × 2 Decomposition of Co-Primary Outcomes

A secondary within-participant factorial decomposition estimated the LA main effect, the CR main effect, and the LA×CR interaction for each co-primary outcome (Table 7). Holm adjustment was applied across the four co-primary outcomes separately within each factorial-effect family.

The Holm-adjusted LA and CR main effects were statistically significant for all four co-primary outcomes. In contrast, none of the LA×CR interactions remained statistically significant after correction (all pHolm ≥ 0.104). The condition-wise advantage of LA+CR for selected outcomes therefore should not be interpreted as evidence of statistical or biological synergy.

These factorial contrasts use treatment-labelled within-participant summaries and do not resolve period, sequence, or first-order carryover effects. The retained analysis dataset did not contain randomized sequence or period identifiers, so a crossover mixed model including those terms could not be fitted. The factorial findings are therefore reported as a secondary sensitivity analysis.

## 4. Discussion

This randomized, double-blind, four-period crossover trial compared acute placebo, creatine monohydrate (CR), L-arginine (LA), and combined LA+CR conditions across repeated-sprint, countermovement jump (CMJ), and Stroop outcomes. The response pattern was outcome-specific rather than uniformly favorable to the combined condition. LA+CR produced the highest RAST peak power (PP) and the greatest CMJ height, with both outcomes exceeding placebo and either single-agent condition. Average power (AP) was higher with LA+CR than with placebo and CR, but the LA+CR versus LA contrast did not remain significant after Bonferroni adjustment. LA alone produced the highest minimum power (MP), whereas fatigue index (FI) did not differ among conditions. For Stroop performance, LA+CR yielded the fastest incongruent reaction time (RT), but it did not differ significantly from LA, and accuracy was unchanged. These findings therefore support selective acute performance differences rather than a generalized combined-supplement advantage.

The data also do not demonstrate biological synergy. The secondary factorial decomposition formally estimated CR×LA interactions for the four co-primary outcomes, and none remained statistically significant after Holm correction (all pHolm ≥ 0.104). Moreover, the combined condition was not consistently superior across endpoints: it was clearly favorable for PP and CMJ in condition-wise comparisons, only partly distinguishable from LA for AP and Stroop RT, and not favorable for MP. Because period and sequence effects could not be modelled in the retained analysis dataset, the factorial estimates should be interpreted as sensitivity contrasts rather than a complete crossover model. This interpretation is consistent with the limited direct literature on combined CR and LA administration [34,35] and with the broader principle that combining two plausible ergogenic agents does not necessarily produce additive effects [8].

The RAST results suggest that the intervention influenced different expressions of anaerobic performance differently. LA+CR increased PP by 99.01 W relative to placebo and also exceeded both single-agent conditions. Creatine is mechanistically relevant to short-duration high-intensity exercise because the creatine-phosphocreatine system supports rapid ATP buffering [23,24]. However, the strongest ergogenic evidence for creatine is based on repeated dosing that increases intramuscular total creatine and phosphocreatine [25,26,27,28,44]. The present CR dose of 0.3 g·kg^−1^ was administered as a single bolus; therefore, the PP response should not be interpreted as proof that muscle phosphocreatine stores increased sufficiently within the pre-test interval to explain the effect. A single-session observation cannot be assumed to reproduce the mechanism of conventional loading.

The AP, MP, and FI findings further argue against a uniform fatigue-resistance effect. Although LA+CR produced the highest mean AP, it did not differ significantly from LA after multiplicity adjustment. LA alone produced the highest MP and exceeded both placebo and LA+CR in selected contrasts, while FI was unchanged. Acute LA studies have likewise produced heterogeneous performance responses [7,48,49]. LA may influence NO production, vascular tone, and ammonia handling [10,14,15,50], but these mechanisms were not measured here. The divergence between PP, MP, and FI therefore indicates that the observed effects were specific to certain power characteristics rather than evidence of a general improvement in repeated-sprint fatigue resistance.

CMJ height showed the largest and most consistent combined-condition response. LA+CR exceeded placebo by 6.72 cm and was also higher than LA and CR alone; both single-agent conditions exceeded placebo but did not differ from each other. Creatine supplementation has been associated with improvements in jumping and high-intensity performance when exposure is sufficient to increase muscle creatine availability [3,27,51]. Nevertheless, those studies cannot establish an identical mechanism for a single acute bolus. NO-related pathways may also influence skeletal-muscle contractile function and perfusion [19,20,21,22], but no vascular or muscle biochemical markers were obtained. The magnitude of the present CMJ difference is substantial for a one-session nutritional intervention and should therefore be replicated before being considered a stable or practice-changing effect.

The CMJ protocol was standardized and demonstrated good reliability, which supports within-participant consistency. Even so, a best-of-three jump outcome may remain sensitive to motivation, learning, and repeated exposure in a four-visit crossover study. Because the Stroop task always preceded CMJ, CMJ also occurred later than the nominal 60-min post-ingestion start time. These factors do not invalidate the result, but they reinforce the need to interpret it within the exact timing and repeated-measures structure of the present protocol.

The Stroop findings indicate a task-specific RT difference rather than broad cognitive enhancement. All active conditions were faster than placebo, and LA+CR produced the lowest mean incongruent RT; however, LA+CR did not differ significantly from LA. Accuracy remained statistically similar and was already very high, suggesting a ceiling effect. A cognitive rationale for creatine is plausible because the brain relies on ATP buffering and cerebral creatine-phosphocreatine metabolism [30,41,42], yet evidence for cognitive benefits in healthy individuals remains mixed [29,31,33]. The absence of a clear LA+CR advantage over LA also argues against attributing the RT finding specifically to an acute cerebral creatine effect.

LA has a plausible relationship with vascular and neurovascular function because oral LA can increase circulating arginine relatively rapidly [52,53], and NO participates in vascular regulation and neurovascular coupling [37]. Nevertheless, the trial did not measure plasma arginine, nitrate/nitrite, cerebral blood flow, or neurophysiological responses. In addition, the Stroop task was administered in English to Turkish-speaking participants, and repeated exposure across four visits may have contributed to practice effects. Accordingly, the cognitive conclusion should remain limited to incongruent Stroop response speed under the tested conditions rather than being generalized to executive function or cognition as a whole [47,54].

The 60-min pre-test interval was chosen because pharmacokinetic studies show relatively rapid increases in circulating L-arginine after oral administration, with peak concentrations occurring around the first hour [52,53]. Short-term hemodynamic effects have also been reported after oral LA [55], although findings from clinical populations cannot be directly extrapolated to healthy recreational athletes. Importantly, only the Stroop task began at approximately 60 min; CMJ and RAST occurred sequentially thereafter. Thus, the three performance domains were not evaluated at an identical post-ingestion time.

The temporal issue is more uncertain for CR because conventional creatine efficacy is primarily supported by repeated loading and maintenance strategies [2,27,44]. Direct evidence on combined LA and CR remains sparse [34,35], and current evidence does not establish that acute LA accelerates muscle or brain creatine uptake. Without plasma arginine, NO-related metabolites, or muscle/brain creatine measurements, the present findings cannot identify the mechanism responsible for the performance pattern. Future mechanistic trials should directly measure exposure and tissue-level intermediates rather than infer them from performance changes alone.

Several features strengthen the study: prospective registration, randomization, double blinding, a within-participant crossover structure, counterbalanced treatment order, standardized testing time, and efforts to control diet and caffeine. Assessing sprint power, explosive jump performance, and interference-control RT also provided a broader view of acute responses than a single endpoint would have offered. These strengths improve within-participant comparability and make the contrasting outcome pattern scientifically informative.

The limitations are nevertheless substantial. First, the retained analysis dataset did not include randomized-sequence or period identifiers, so period, sequence, treatment-by-period, and first-order carryover effects could not be estimated. In addition, the ≥72-h washout may be insufficient to exclude creatine-related carryover. After conventional loading, elevated muscle creatine can persist for considerably longer than several days [44]. A single bolus may produce less accumulation, but no muscle creatine measurement was available to verify return to baseline. Second, Holm correction controlled the family-wise error rate across the four co-primary omnibus tests and separately across each factorial-effect family, while post hoc tests were Bonferroni-adjusted within outcome. Nevertheless, the original sample-size calculation was not multiplicity-adjusted, and multiplicity was not controlled across the entire collection of secondary and exploratory analyses. The findings should therefore be regarded as exploratory.

Third, the placebo was matched for volume, flavor, and appearance but was not mass-matched or iso-osmotic, and blinding efficacy was not formally assessed. Fourth, no plasma arginine, nitrate/nitrite, muscle creatine/phosphocreatine, or related mechanistic biomarkers were measured. Fifth, dietary and caffeine compliance relied partly on self-report, and habitual pre-study creatine/arginine exposure was not characterized in sufficient detail. Sixth, the sample was small, male-only, recreationally active, and drawn from a single center, limiting generalizability. Large within-subject standardized effects also require caution because paired standardized effects can become large when the variability of within-person differences is small [56]. Finally, the fixed Stroop → CMJ → RAST sequence and repeated exposure may have introduced practice or task-order effects. Although no corrected CR×LA interaction was detected, the absence of a significant interaction does not prove additivity, synergy, or antagonism, particularly when period and sequence effects remain unmodelled.

From an applied perspective, the PP, CMJ, and Stroop RT differences are potentially relevant to activities requiring explosive actions and rapid information processing, but the present data are insufficient to recommend acute LA+CR co-supplementation for competition or routine training. Athletes should not interpret the results as showing that a single large creatine dose approximately 60 min before exercise is an established alternative to conventional creatine-loading or maintenance strategies [44]. A confirmatory study should prespecify one primary outcome, use a sample size and multiplicity plan based on that endpoint, employ a pharmacokinetically justified washout or a parallel-group design, formally test CR × LA interaction effects, assess blinding and tolerability, and include sex-inclusive samples with mechanistic biomarkers.

Overall, acute LA+CR was associated with favorable changes in selected high-intensity and task-specific outcomes, especially PP and CMJ, but the evidence does not establish a unified mechanism or a general performance advantage. The main contribution of the present study is therefore hypothesis generation: it identifies specific signals that warrant targeted replication in designs capable of resolving carryover, interaction effects, and biological mediation.

## 5. Conclusions

In this randomized, double-blind crossover study of recreationally active men, acute LA+CR administration produced the most favorable mean responses for RAST peak power, CMJ height, and incongruent Stroop reaction time; however, the pattern was not uniform across outcomes. LA+CR was superior to both single-agent conditions for peak power and CMJ height, whereas its advantage over LA was not statistically significant for average power or Stroop reaction time after within-outcome adjustment. Holm correction across the four co-primary outcomes did not alter the significance of their omnibus condition effects, but none of the LA×CR interactions remained significant after Holm correction. Accordingly, the findings support outcome-specific acute responses rather than a general ergogenic effect or evidence of biological synergy between CR and LA. These results should remain preliminary because the sample was small and male-only, the sample-size calculation was not multiplicity-adjusted, period and sequence effects could not be modelled, a single creatine bolus is not an established acute loading strategy, and the ≥72-h washout cannot be assumed to eliminate creatine-related carryover. The data therefore do not yet justify practical recommendations for acute LA+CR co-supplementation. Confirmation is needed in larger, sex-inclusive studies using pharmacokinetically justified washout periods, prospectively specified multiplicity plans, retained period/sequence data, matched placebo conditions, formal CR×LA interaction analyses, and mechanistic measures such as circulating arginine-related markers and muscle creatine/phosphocreatine status.

## Figures and Tables

**Table 1:** Participant characteristics (N = 18 males).

Variable	Mean ± SD	Min	Max
Age (years)	21.4 ± 2.1	18	27
Height (cm)	177.3 ± 4.8	167	184
Body mass (kg)	71.8 ± 10.1	54	87
BMI (kg/m^2^)	22.8 ± 2.9	18.2	27.2

**Table 2:** Descriptive statistics (mean ± SD) for all outcome variables across four conditions (N = 18).

Variable	CG	LA	CR	LA+CR
AP (W)	447.34 ± 65.71	480.16 ± 74.89	480.24 ± 75.65	494.19 ± 70.36
PP (W)	494.83 ± 70.49	542.54 ± 91.90	547.13 ± 103.95	593.83 ± 85.97
MP (W)	409.02 ± 67.60	435.41 ± 71.14	420.36 ± 67.34	387.16 ± 82.68
FI (%)	21.66 ± 7.11	24.74 ± 8.72	22.38 ± 8.13	21.84 ± 4.79
CMJ (cm)	33.92 ± 4.53	37.24 ± 4.42	37.97 ± 4.27	40.64 ± 3.88
Stroop RT (s)	0.626 ± 0.105	0.565 ± 0.093	0.557 ± 0.087	0.537 ± 0.080
Stroop Acc (%)	97.33 ± 1.75	98.17 ± 1.82	98.17 ± 1.51	97.89 ± 1.91

AP = average power; PP = peak power; MP = minimum power; FI = fatigue index; CMJ = countermovement jump; RT = reaction time; Acc = accuracy.

**Table 3:** One-way repeated-measures ANOVA results (N = 18). GG ε = Greenhouse–Geisser epsilon (applied when Mauchly’s test was significant); — = sphericity assumed.

Outcome	df_1_	df_2_	F	*p*	ηp^2^	GG ε
AP (W)	1.92	32.70	26.56	<0.001	0.610	0.641
PP (W)	3.00	51.00	24.35	<0.001	0.589	—
MP (W)	1.89	32.15	5.37	0.011	0.240	0.630
FI (%)	1.71	29.03	1.22	0.306	0.067	0.569
CMJ (cm)	3.00	51.00	85.36	<0.001	0.834	—
Stroop RT (s)	1.95	33.21	15.84	<0.001	0.482	0.651
Stroop Acc (%)	3.00	51.00	1.01	0.396	0.056	—

**Table 4:** Pairwise comparisons for RAST outcomes (N = 18). MD = mean difference (W). *p* values are Bonferroni-adjusted within outcome; 95% CIs are unadjusted and should therefore not be used as multiplicity-adjusted significance tests.

Comparison	AP:MD [95% CI]	*p*	PP:MD [95% CI]	*p*	MP:MD [95% CI]	*p*
LA+CR vs. CG	46.85 [32.82, 60.87]	<0.001	99.01 [74.19, 123.83]	<0.001	−21.86 [−55.33, 11.60]	1.000
LA+CR vs. LA	14.02 [3.92, 24.12]	0.056	51.29 [26.66, 75.93]	0.002	−48.25 [−79.38, −17.13]	0.027
LA+CR vs. CR	13.95 [5.54, 22.35]	0.016	46.71 [27.25, 66.16]	<0.001	−33.20 [−63.13, −3.27]	0.190
LA vs. CG	32.82 [20.30, 45.34]	<0.001	47.71 [23.45, 71.98]	0.004	26.39 [16.45, 36.33]	<0.001
CR vs. CG	32.90 [17.85, 47.95]	0.002	52.30 [21.05, 83.56]	0.015	11.33 [−12.33, 35.00]	1.000
LA vs. CR	0.08 [−6.44, 6.59]	1.000	4.59 [−16.02, 25.19]	1.000	15.06 [−6.63, 36.75]	0.968

**Table 5:** Pairwise comparisons for Stroop incongruent RT (N = 18). d_x_ = within-subject standardized mean difference. *p* values are Bonferroni-adjusted within outcome; 95% CIs are unadjusted.

Comparison	MD (s)	SE	95% CI	*p*	d_x_
CG vs. LA+CR	0.089	0.013	[0.057, 0.121]	<0.001	1.38
CG vs. LA	0.061	0.013	[0.033, 0.089]	0.002	1.08
CG vs. CR	0.069	0.016	[0.032, 0.106]	0.006	0.93
CR vs. LA+CR	0.020	0.006	[0.006, 0.034]	0.041	0.73
LA vs. LA+CR	0.028	0.012	[0.001, 0.056]	0.271	0.51
LA vs. CR	0.008	0.012	[−0.021, 0.037]	1.000	0.14

**Table 6:** Pairwise comparisons for CMJ height (N = 18). d_x_ = within-subject standardized mean difference. *p* values are Bonferroni-adjusted within outcome; 95% CIs are unadjusted.

Comparison	MD (cm)	SE	95% CI	*p*	d_x_
LA+CR vs. CG	6.72	0.412	[5.76, 7.69]	<0.001	3.47
LA+CR vs. LA	3.40	0.349	[2.59, 4.21]	<0.001	2.08
LA+CR vs. CR	2.67	0.304	[1.96, 3.38]	<0.001	1.87
LA vs. CG	3.32	0.433	[2.31, 4.34]	<0.001	1.63
CR vs. CG	4.05	0.318	[3.31, 4.79]	<0.001	2.71
LA vs. CR	−0.73	0.451	[−1.78, 0.33]	0.983	0.34

Note: within-subject d_x_ values can be large when the SD of paired differences is small; practical interpretation should prioritize the raw MDs and their precision. The 95% CIs shown are unadjusted, whereas *p* values are Bonferroni-adjusted.

**Table 7:** Secondary 2 × 2 factorial contrasts for the four co-primary outcomes (N = 18). Values are mean contrast [95% CI]; pHolm. Holm adjustment was applied across the four co-primary outcomes separately for the LA main-effect, CR main-effect, and LA×CR interaction families. Lower Stroop RT values indicate faster responses.

Outcome	LA Main Effect	CR Main Effect	LA×CR Interaction
AP (W)	23.38 [17.51, 29.26]; <0.001	23.46 [14.24, 32.69]; <0.001	−18.88 [−36.67, −1.08]; 0.117
PP (W)	47.21 [36.91, 57.51]; <0.001	51.80 [31.44, 72.15]; <0.001	−1.01 [−39.87, 37.86]; 0.957
CMJ (cm)	3.00 [2.23, 3.77]; <0.001	3.73 [3.07, 4.38]; <0.001	−0.65 [−1.49, 0.19]; 0.243
Stroop RT (s)	−0.041 [−0.054, −0.027]; <0.001	−0.048 [−0.076, −0.021]; 0.002	0.041 [0.006, 0.076]; 0.104

## Data Availability

The data presented in this study are available from the corresponding author upon reasonable request.
